# Molecular Adsorption of NH_3_ and NO_2_ on Zr and Hf Dichalcogenides (S, Se, Te) Monolayers: A Density Functional Theory Study

**DOI:** 10.3390/nano10061215

**Published:** 2020-06-22

**Authors:** Shimeles Shumi Raya, Abu Saad Ansari, Bonggeun Shong

**Affiliations:** Department of Chemical Engineering, Hongik University, Seoul 04066, Korea; shimtg2011@gmail.com

**Keywords:** transition metal dichalcogenide, TMDC, molecular adsorption, charge transfer, band gap

## Abstract

Due to their atomic thicknesses and semiconducting properties, two-dimensional transition metal dichalcogenides (TMDCs) are gaining increasing research interest. Among them, Hf- and Zr-based TMDCs demonstrate the unique advantage that their oxides (HfO_2_ and ZrO_2_) are excellent dielectric materials. One possible method to precisely tune the material properties of two-dimensional atomically thin nanomaterials is to adsorb molecules on their surfaces as non-bonded dopants. In the present work, the molecular adsorption of NO_2_ and NH_3_ on the two-dimensional trigonal prismatic (1H) and octahedral (1T) phases of Hf and Zr dichalcogenides (S, Se, Te) is studied using dispersion-corrected periodic density functional theory (DFT) calculations. The adsorption configuration, energy, and charge-transfer properties during molecular adsorption are investigated. In addition, the effects of the molecular dopants (NH_3_ and NO_2_) on the electronic structure of the materials are studied. It was observed that the adsorbed NH_3_ donates electrons to the conduction band of the Hf (Zr) dichalcogenides, while NO_2_ receives electrons from the valance band. Furthermore, the NO_2_ dopant affects than NH_3_ significantly. The resulting band structure of the molecularly doped Zr and Hf dichalcogenides are modulated by the molecular adsorbates. This study explores, not only the properties of the two-dimensional 1H and 1T phases of Hf and Zr dichalcogenides (S, Se, Te), but also tunes their electronic properties by adsorbing non-bonded dopants.

## 1. Introduction

Nanomaterials often manifest fascinating and useful properties, which can be exploited for a variety of applications [1,2,3,4,5,6,7,8,9]. For example, electronic devices are miniaturized to nanoscale. However, this development faces some issues, such as replacing the currently used SiO_2_ gate oxides of complementary metal–oxide–semiconductor (CMOS) transistors with another high-k material [10]. Also, for sub-10-nm field-effect transistors (FETs), effective gate control is needed. Furthermore, Si suffers from surface roughness (SR) effects that can reduce their charge carrier mobility [11] and lead to strong variability in threshold voltages [12]. Encouragingly, in recent years, the introduction of high-k gate dielectrics and metal gates has been successful for improving transistor performance [13]. However, the current International Technology Roadmap for Semiconductors (ITRS) predicts that, to fulfill the expected demand for nanodevices, novel materials with extreme properties will be needed to successfully address the challenges of transistor scaling in the next decade [14]. A current focus of nanotechnology is on atomically thin semiconductor materials. The use of two-dimensional (2D) materials enables nano-scale transistors without dangling bonds. However, new challenges exist, such as bandgap, non-negligible contact resistance, and the difficulty in integrating high-k gate insulators with most 2D materials. The fact that the large bandgap (E_g_ = 9 eV) of SiO_2_ and its high-quality interface with Si enables the isolation of Si components and a reduction of additional gate leakage currents is noteworthy. Thus, if one wants to replace Si in these materials, the candidate material must not only demonstrate properties similar to Si, but also, their native oxides should exhibit high dielectric constants.

Two-dimensional transition metal dichalcogenides (TMDCs) are gaining research interest due to their atomic thickness and unique mechanical, electric, and optical properties, further, they are considered as promising high-performance electronic and optoelectronic materials [15,16]. Depending on their chemical compositions and structural configurations, 2D TMDC materials can be categorized as metallic, semimetallic, semiconducting, insulating, or superconducting. A semimetal exhibits the feature whereby a small overlap exists between the top of the valance band and the bottom of the conduction band. For example, some group-IVB TMDCs show semimetal features due to a small overlap between the top of the p-orbital chalcogen valance band and the bottom of the d-orbital transition metal conduction band [17]. Many 2D TMDCs are semiconductors by nature, and possess a huge potential to be made into ultra-small and low-power transistors that are more efficient than state-of-the-art silicon-based transistors fighting to cope with ever-shrinking devices [16]. Semiconducting TMDCs have advantages over gapless graphene in applications for logic transistors, photodetectors, and FETs, since a sizable bandgap is necessary to achieve high on/off ratio, which these materials possess [16]. The most widely investigated semiconducting TMDC, MoS_2_, depicts good mobility (~100 cm^2^∙V^−1^∙s^−1^ in sub-2-nm-thick films) independent of channel thickness and a high on/off FET current ratio (~10^6^) near room temperature [18,19]. Furthermore, MoS_2_ does not exhibit a large SR and is thus advantageous to be used in place of Si in sub-10-nm FETs.

Conversely, Zr- and Hf-based TMDCs demonstrate a moderate bandgap comparable to Si. Furthermore, they demonstrate the unique advantage that their native oxides (ZrO_2_ and HfO_2_) are excellent dielectric materials, which show potential to replace Si in semiconductor technology [20]. These TMDCs exhibit ohmic contact like Si with their native oxides, which enable the isolation of components, and they demonstrate a reduced leakage current compared to Si transistors. Although, Mo- and W-based TMDCs and their native oxides (MoO_3_ and WO_3_) depict similar features, MoO_3_ and WO_3_ are not good insulators, and they may even act as dopants [21,22,23].

The electronic and optoelectronic properties of present TMDC materials are sometimes not good enough, and additional candidate TMDC materials are being sought. So far, 2D semiconducting Zr- and Hf-based TMDCs from group IVB were not investigated as much as their counterparts from group VIB. Further, changing the chalcogen species (S, Se, Te) in TMDCs can trigger paradigm changes to their electronic structure, and in turn alter their electronic and optoelectronic attributes. Recently, Zr- and Hf-based TMDCs were theoretically predicted to exhibit higher mobilities and higher sheet current densities than group-VIB (Mo and W) TMDCs [14,24]. Inspired by this, 2D HfS_2_, HfSe_2_, and ZrS_2_ were studied for their potential applications in FETs and phototransistors [20,25,26]. However, further investigations are needed to shed light on 2D Zr- and Hf-based TMDCs for their potential applications, and new findings in nanoscience are subsequently anticipated.

In addition to implementing 2D materials in nanodevices, tuning of the material properties of 2D materials is very important. One possible method to precisely tune the material properties of 2D atomically thin nanomaterials is to adsorb molecules on their surfaces as non-bonded dopants [27]. Researchers demonstrated that the molecular adsorption of NO_2_, NH_3_, H_2_O, CO, borazine, triazine, and benzene on gapless graphene led to the band gap widening due to the adsorption-site-dependent magnitude of the band gap [28,29]. Such phenomenon is seen in the present investigation after NH_3_ adsorption. The molecular adsorption of NO_2_ and NH_3_ on 2D MoS_2_ was also studied by Luo et al. [30]. Adsorbing molecules demonstrate the potential to modify the electronic properties, which could be relevant for ultra-small low-power electronic devices. The adsorbing molecules serve as either an electron donor or acceptor, thereby producing a temporary charge perturbation in the adsorbing material. To date, no such study on 2D Zr- and Hf-based TMDCs was conducted.

In the present work, the molecular adsorption of NH_3_ and NO_2_ on 2D Zr and Hf dichalcogenides (S, Se, Te) are studied using density functional theory (DFT) calculations. The adsorption configuration, energy, and charge-transfer properties during molecular adsorption are calculated. In addition, the effects of the molecular dopants (NH_3_ and NO_2_) on the electronic structure of the materials are studied. Researchers observed that adsorbed NH_3_ donates electrons to the conduction band of the Zr (Hf) dichalcogenides, while NO_2_ received electrons from the valance band. The resulting band structure of the molecularly doped Zr and Hf dichalcogenides are modulated by the molecular adsorbates. Therefore, by introducing molecular dopants such as NH_3_ and NO_2_ to TMDCs, we confirm that the material properties of these substrates can be tuned.

## 2. Computational Methods

**Figure 1:**
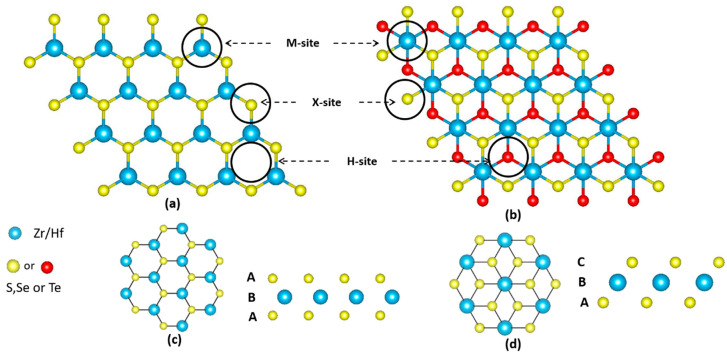
2D Zr (Hf) dichalcogenides, (**a**) trigonal prismatic (1H) and (**b**) octahedral (1T) sheet structure, where blue color represents metal (Zr or Hf) and yellow (red) color represents chalcogen (S, Se or Te); (**c**) 1H arrangement (ABA staking), (**d**) 1T arrangement (ABC staking).

The DFT calculations, using slab models, were performed using Vienna ab initio simulation package (VASP) version 5.4.4 [31], employing Perdew-Burke-Ernzerhof (PBE) exchange−correlation functionals [32] and the projector-augmented wave method [33]. Dispersion interactions were considered via the Grimme method [34]. To optimize the lattice parameters of the metal dichalcogenides (MX_2_, M = Zr, Hf, and X = S, Se, Te), their unit cell structures were fully relaxed using the conjugate gradient method [35] until the maximum Hellmann–Feynman force acting on each atom was less than 0.02 eV∙A^−1^. The optimized lattice parameters are close to the reported values (Table 1), which, in later sections, will be discussed in detail. 4 × 4 × 1 trigonal prismatic (1H) and octahedral (1T) supercells containing 48 atoms were constructed using the calculated lattice parameters, which were employed to simulate the pristine 2D Zr (Hf) dichalcogenides (S, Se, Te). A spacing of 20 Å in the vertical direction was added to minimize any unphysical interactions between the slabs. A (5 × 5 × 1) gamma (γ) k-point mesh and an energy cutoff of 500 eV were used after testing the slab-energy convergence. Three different sites on the 1H and 1T supercell Zr (Hf) dichalcogenides, namely, T_M_ (top of metal), T_X_ (top of chalcogen), and T_H_ (top of hexagon), were considered for NH_3_ (NO_2_) adsorption, as shown in Figure 1. Two orientations of the adsorbing gas molecules, i.e., the hydrogen (oxygen) atom of NH_3_ (NO_2_) oriented away from the adsorbent surface (U-orientation) or toward the surface (D-orientation) were considered. The adsorbates and the slab were allowed to relax until the residual force became less than 0.02 eV∙Å^−1^.

To characterize the interaction strength between the adsorbate gas molecule and the adsorbent material, the molecular adsorbates electronic binding energy (*E_binding_*) was obtained using the following equation [36],

(1) $$E_{binding}=E_{total}-E_{surface}-E_{adsorbate}$$

where *E_total_*, *E_surface_*, and *E_adsorbate_* are the total electronic energies of the slab with adsorbates, the pristine Zr (Hf) dichalcogenides slab, and the free adsorbates, respectively. By means of a Bader analysis [37], the charge transfer between the monolayer substrate and the adsorbate was obtained. The adopted dispersion-corrected method is very accurate for structural and adsorption energy calculations. However, even though the bandgap values are underestimated, this method still yields reasonably correct features of electronic structure. Further, the charge density difference (Δ*ρ*) was defined to be consistent with previous calculations of a gas on a surface [35], which is calculated as $∆\rho =\rho_{MX_{2}+gas}-\left(\rho_{MX_{2}}+\rho_{gas}\right)$, where $\rho_{MX_{2}+gas}$, $\rho_{MX_{2}}$, and $\rho_{gas}$ are the charge density of the gas-molecule-adsorbed TMDC surface, the pristine TMDC surface, and the isolated gas molecules, respectively.

## 3. Results and Discussion

Prior to NH_3_ (NO_2_) adsorption, the lattice parameters (*a*) of the 1T and 1H Zr (Hf) dichalcogenides were calculated. To the best of our knowledge, only the 1T structure has been taken into consideration. To date, no lattice parameter data, either theoretical or experimental, that are related to 1H Zr (Hf) dichalcogenides, were published and made available. Hence, in this work, the 1H structure lattice parameters are discussed by comparisons with the 1T structure. Table 1 summarizes the optimized lattice parameters (Å) of the 2D 1T and 1H Zr (Hf) dichalcogenides. The lattice parameters of 1H are slightly smaller in all cases, as compared to the 1T Zr (Hf) dichalcogenides, which are associated with a decrease in the ionic radius of S. This is due to differences in atom stacking: 1H possess an ABA-type atomic staking, while 1T demonstrates an ABC-type atomic staking. As the atomic indices change from Te to Se and from Se to S, the lattice constants and metal-to-chalcogen bond lengths decrease due to the decreased atomic radius of the chalcogen. When switching from 1T to 1H phases, similar changes occur. The maximum difference in the calculated lattice parameters of the 1H and 1 T structure is found for HfS_2_ (3.04%), whereas a minimum is found for ZrTe2 (0.77%); we find the overall order HfS_2_ > ZrS_2_ > HfSe_2_ > ZrSe_2_ > HfTe_2_ > ZrTe_2_. The calculated lattice parameters of the 1T-structured Zr (Hf) dichalcogenides are in close agreement with reported theoretical and experimental lattice parameters [38,39,40,41]. No experimental data are available for the lattice parameter of 1T-HfTe_2_ and 1T-ZrTe_2_. The variation of lattice parameters from previous theoretical data is due to the experimental conditions and the level of theory used for the calculations.

**Figure 2:**
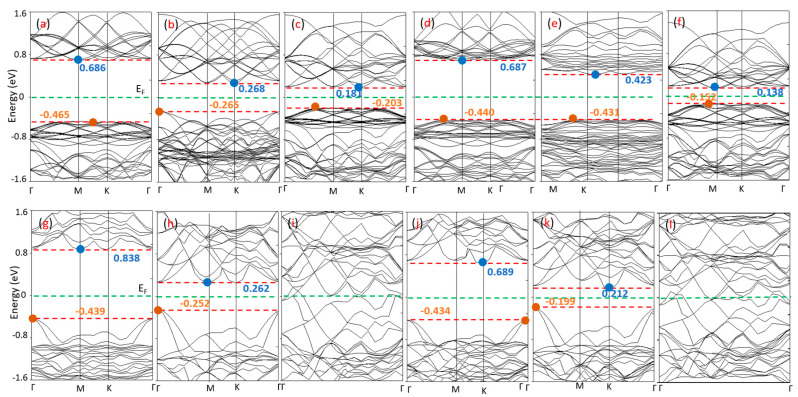
Band structures of pristine 2D Zr (Hf) dichalcogenides, (top) 1H and (bottom) 1T: [(**a**),(**g**)] HfS_2_, [(**b**),(**h**)] HfSe_2_, [(**c**),(**i**)] HfTe_2_, [(**d**),(**j**)] ZrS_2_, [(**e**),(**k**)] ZrSe_2_, and [(**f**),(**l**)] ZrTe_2_.

To investigate their electronic properties, we focused on the band structures of the 1T and 1H Zr (Hf) dichalcogenides and compared 1T relative to 1H in terms of their conduction bands, valance bands, and band gaps. Figure 2 depicts the band structures of the pristine 2D 1T and 1H Zr (Hf) dichalcogenides obtained by the PBE method. The band structures show that they are indirect band gap semiconductors, similar to corresponding bulk TMDCs. This feature is different to that seen for some other TMDCs, such as MoS_2_ or WS_2_. The bulk MoS_2_ and WS_2_ systems show indirect bandgap transitions, but they gradually shift to direct transitions for the monolayer [15]. Further, 1T-HfTe_2_ and 1T-ZrTe_2_ show semimetallic features instead of semiconductor features, which is consistent with earlier experimental [42] and theoretical [40,43] reports.

Table 2 summarizes the calculated band gaps of the pristine 2D 1T and 1H Zr (Hf) dichalcogenides (S, Se & Te) [39,40,41,43,44]. To date, no reports are available on the band structure and band gap of 1H Zr (Hf) dichalcogenides.

Researchers reported theoretically that in 1T-HfS_2_, the Hf–d and S–p states are located between −5 eV and the Fermi energy (E_F_) [40], whereas, the bands from E_F_ to 2.4 eV (~ 3 eV for 1H-HfS_2_) consist of Hf–d and Hf–f states with a small contribution from the S–p state [40]. The valance band maximum (VBM) is observed at Γ, which is consistent with the calculations of Murray et al. [45], Mattheis [46], Fong et al. [47], and Reshak and Auluck [40]. The conduction band minimum (CBM) is located at M, in agreement with the calculations of Fong et al. [47] and Traving et al. [48]. Further, researchers reported theoretically that a strong hybridization exists between the Hf–f and Hf–d states below and above E_F_, respectively, and a weak hybridization exists between the Hf–d and S–p states below E_F_ [40]. Conversely, the 1H-HfS_2_ band gap (1.15 eV) is slightly smaller than that of 1T-HfS_2_ (1.28 eV). The conduction band slightly shifts to a lower value of 0.15 eV. Further, we find that the energy band structures pattern of 1H-HfS_2_ is different to that of 1T-HfS_2_.

The band structure of 1T-HfSe_2_ is similar to that of 1T-HfS_2_, but it exhibits a smaller energy gap (Table 2). Replacing S with Se causes a separation of the Hf–f states from the Hf–d states below and above E_F_ [40]. Further, the hybridization below E_F_ between Hf–d and Se–p is stronger than in 1T-HfS_2_. Identical to 1T-HfS_2_, the VBM is located at Γ, and the CBM is at M, in agreement with Murray et al. [45]. Although, the band structures of 1H and 1T HfSe_2_ differ from each other, the positions of the valance and conduction bands are identical, and hence both show nearly the same band gap value.

In 1T-ZrS_2_, the VBM is located at Γ, while the CBM is located between Γ and K, resulting in an indirect gap of 1.13 eV. It is known theoretically that the band between −5 eV to E_F_ and E_F_ to 3 eV is composed of Zr-d and a small contribution from S-p states [43], as well as a strong hybridization between Zr-d and S-p states below E_F_ [43]. The 1T-ZrS_2_ band looks similar to that of 1T-HfS_2_ but with a slightly decreased bandgap. Conversely, the 1H-ZrS_2_ bands not only match those of 1H-HfS_2_, but they also show nearly the same electronic gap. However, the 1H-ZrS_2_ band structure is quite different to that of 1T-ZrS_2_, although they demonstrate nearly the same bandgap (Table 2) with identical band positions.

In 1T-ZrSe_2_, the VBM is located at Γ, and the CBM is at M [43,45]. With some minor differences, the band structure of 1T-ZrSe_2_ is like that of 1T-ZrS_2_, such as the reduction in the bandwidth of the Se-S group that is shifted toward lower energies with respect to E_F_ along with a second group band enhancement [43]. Furthermore, the shift in the conduction bands at about 0.5 eV toward lower energies leads to 1T-ZrSe_2_, demonstrating a smaller energy gap than 1T-ZrS_2_. In contrast, no shifting and enhancement of second group band toward EF in 1H-ZrSe_2_ than 1H-ZrS_2_. Additionally, the conduction band in 1T-ZrS_2_ shifts toward E_F_ less than that of 1T-ZrSe_2_. Thus, 1H-ZrSe_2_ possesses a higher band gap (0.85 eV) than 1T-ZrSe_2_ (0.41 eV).

In 1T-ZrTe_2_ (1T-HfTe_2_), the occupied and unoccupied bands move toward E_F_, which closes the energy gap and indicates metallic behavior. Also, a strong hybridization exists between the Te–p and Zr (Hf) d states below E_F_ [40,43]. Interestingly, 1H-ZrTe_2_ (1H-HfTe_2_) still shows semiconducting behavior, although its energy gap is lower than that of 1H-ZrSe_2_ (1H-HfSe_2_). A rise in the conduction band and fall in the valance band toward E_F_ is clearly observed.

It is to be noted that the Eg value of ZrS_2_ (HfS_2_) is a maximum, and it decreases as we replace chalcogen S with first Se and then Te. Although the amount of decrease in the Eg value in the Zr dichalcogenides differs to that of the Hf dichalcogenides, their decreasing trends are similar. The band structure of the Zr dichalcogenides looks similar to that of the Hf dichalcogenides, except for the case of 1H-ZrSe_2_ and 1H-HfSe_2_ (where they look different), even though their Eg values differ. Furthermore, both 1T-HfTe_2_ and 1T-ZrTe_2_ depict semimetallic natures. In the 1H structure, as S is replaced by Se, a large reduction occurs in the Eg value for the Hf-based TMDCs relative to the Zr-based TMDCs, while the situation is reversed for materials containing 1T structures. Also, it is well-known that 1T-ZrTe_2_ (1T-HfTe_2_) possesses a semimetallic nature. However, in the case of 1H structures, both materials show band openings; 1H phase Zr(Hf) dichalcogenides depict a band gap energy ranging from 0.29–1.15 eV, while in the 1T phase, the range is 0.41–1.29 eV. These moderate Eg values are comparable to other semiconductor materials such as Si (Eg = 1.14 eV), Ge (Eg = 0.67 eV), and PbS/Se/Te (Eg = 0.37/0.27/0.29 eV) [49]. A semiconductor of this nature can lead to the use of these materials in different applications.

Thereafter, NH_3_ and NO_2_ adsorption on the trigonal prismatic (1H) and octahedral (1T) phases was carried out. First, we optimized the atomic geometries of the NH_3_ and NO_2_ gas molecules using the DFT approach. Based on our calculated results, the bond lengths of the NH bonds of the NH_3_ molecule and the NO bonds of the NO_2_ molecule are 1.02, and 1.21 Å, respectively. These are in reasonable agreement with previously reported data [50,51]. Moreover, the structure of the considered 4 × 4 × 1 supercell of Zr (Hf) dichalcogenides monolayer was geometrically optimized.

**Figure 3:**
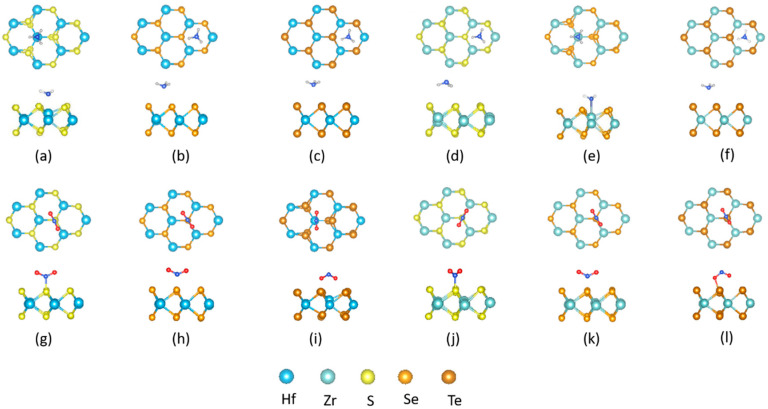
Adsorption configurations and orientations of NH_3_ (top row) and NO_2_ (bottom row) on chemically stable site of 1H: [(**a**),(**g**)] HfS_2_ [(**b**),(**h**)] HfSe_2_ [(**c**),(**i**)] HfTe_2_ [(**d**),(**j**)] ZrS_2_ [(**e**),(**k**)] ZrSe_2_ [(**f**),(**l**)] ZrTe_2_ monolayer sheet.

To search for the most stable configuration of the NH_3_ (NO_2_) molecules on the Zr (Hf) dichalcogenide monolayer, various adsorption positions were examined. For each adsorption configuration, an initial, reasonable distance between the gas molecules and the substrate was chosen. Figure 3 depicts the typical optimized geometry configurations of the NH_3_- and NO_2_-adsorbed 1H Zr (Hf) dichalcogenide monolayer. Configurations a–f represents the adsorption of NH_3_ molecules on the 1H Zr (Hf) dichalcogenides, while configurations g–l shows the interaction between the NO_2_ gas molecules and the 1H Zr (Hf) dichalcogenide monolayer. H-upward (U-orientation) is the preferable configuration for the NH_3_ molecules, except for the case of 1H-ZrS_2_, where H is oriented toward the adsorbing surface. Furthermore, the T_M_-site is preferred for NH_3_ adsorption on 1H-HfS_2_ and 1H-ZrSe_2_, where the adsorption-distances (d; i.e., the shortest atom-to-atom distance between the gas molecules and the substrate) are 2.43 and 2.49 Å (Table 3), respectively, suggesting NH_3_ adsorbs more strongly on 1H-HfS_2_ than 1H-ZrSe_2_. The remaining 1H Zr (Hf) dichalcogenides adsorb NH_3_ on the T_H_-sites with minimum adsorption-distances on HfSe_2_ (3.38 Å). Interestingly, for all cases, no NH_3_ adsorption takes place on the chalcogen sites. In contrast, NO_2_ preferably adsorbs only on T_X_-sites with D-orientation on HfTe_2_ and ZrTe_2_, with the same adsorption distance (2.28 Å). The U-oriented NO_2_ adsorbs on the remaining 1H Zr (Hf) dichalcogenides, with a minimum adsorption-distance of 2.02 Å on 1H-HfS_2_. Conversely, the NH_3_ (NO_2_) molecules adsorb differently on the 1T Zr (Hf) dichalcogenides.

**Figure 4:**
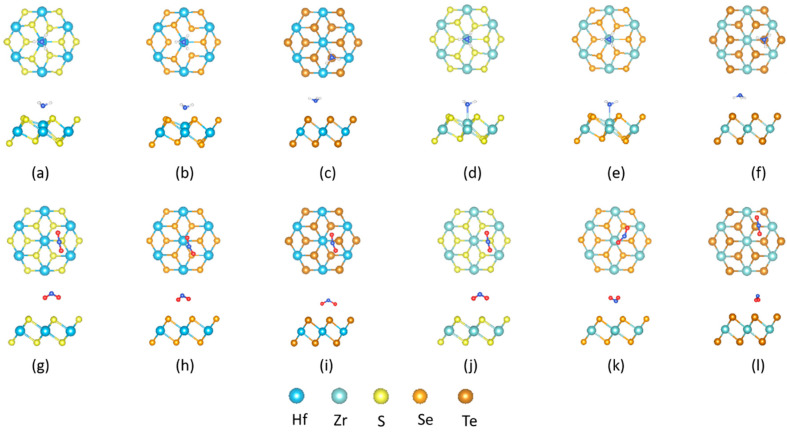
Adsorption configurations and orientations of NH_3_ (top row) and NO_2_ (bottom row) on chemically stable site of 1T: [(**a**),(**g**)] HfS_2_ [(**b**),(**h**)] HfSe_2_ [(**c**),(**i**)] HfTe_2_ [(**d**),(**j**)] ZrS_2_ [(**e**),(**k**)] ZrSe_2_ [(**f**),(**l**)] ZrTe_2_ monolayer sheet.

The optimized geometric configurations of the molecularly adsorbed NH_3_ and NO_2_ on the 1T Zr (Hf) dichalcogenides monolayers are summarized in Figure 4, where configurations a–f and g–l represents the adsorption of NH_3_ and NO_2_ molecules on the 1T Zr (Hf) dichalcogenides, respectively. As compared to 1H, the U-orientation is favorable for NH_3_ on the 1T Zr (Hf) dichalcogenides except for 1T-ZrTe_2_, where the D-orientation is favorable. The largest adsorption-distance is 3.87 Å (Table 4) for NH_3_ on the 1T Zr (Hf) dichalcogenides. The D-orientation (in 1H) changes to U-orientation for NH_3_ on 1T-ZrS_2_. Additionally, the T_M_-site is preferred over the T_H_-site, which is the opposite situation compared with what was observed for the 1H Zr (Hf) dichalcogenides. Consistent with 1H, HfS_2_ shows a minimum NH_3_ adsorption-distance (2.42 Å). A fresh, no preference adsorption takes place on the chalcogen sites. Conversely, the D-orientation is favorable for NO_2_ on the 1T Zr (Hf) dichalcogenides, differing from the previous three cases, with a minimum adsorption-distance of 3.21 Å on 1T-ZrTe_2_, which is almost 1 Å larger than that found for 1H-ZrTe_2_. Furthermore, the T_M_ and T_X_-sites are equally favorable. The details are given in Table 4. It is distinctly seen that in all four cases, the D-orientation exhibits a larger adsorption distance than the corresponding U-orientation. The smallest adsorption-distance on metal sites is found for NH_3_ on 1T-HfS_2_ (2.42 Å) and 1T-ZrS_2_(2.44 Å), which are larger than the sum of the covalent atomic radii of Hf-N (2.26 Å) and Zr-N (2.28 Å). Similarly, the smallest adsorption distance on the chalcogen is found for NO_2_ on 1H-HfS_2_ (2.02 Å), 1H-ZrSe2 (2.17 Å), and 1H-HfTe2(1H-ZrTe2) (2.28 Å), which are significantly larger than experimental average bond lengths of S-N (1.71 Å), Se-N (1.82 Å), and Te-N (2.02 Å) [52]. Thus, no chemical bonds are expected to form, and only the physisorption of NH_3_ and NO_2_ takes place on the 1H and 1T Zr (Hf) dichalcogenides.

**Figure 5:**
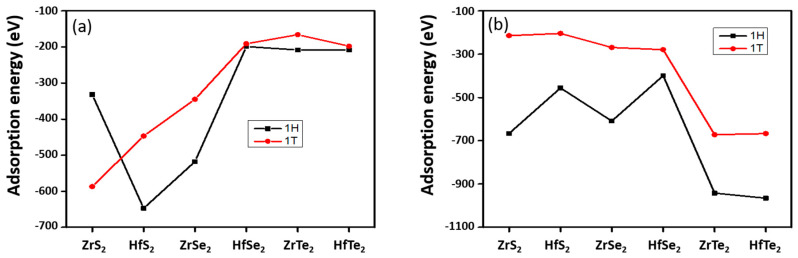
Adsorption energy comparison of (**a**) NH_3_ (**b**) NO_2_ on trigonal prismatic (1H) and octahedral (1T) 2D Zr (Hf) dichalcogenides.

A negative adsorption energy implies that the adsorption of the NH_3_ (NO_2_) molecules on the 1H and 1T Zr (Hf) dichalcogenides is energetically favorable [53]. The adsorption energy (E_ads_) of NH_3_ and NO_2_ on the 1H and 1T Zr (Hf) dichalcogenides at different sites (H-site, T_M_-site, T_X_-site) was calculated and plotted, as shown in Figure 5. In case of the 1H Zr (Hf) dichalcogenides, the largest calculated NH_3_ adsorption energy is −647 meV on 1H-HfS_2_, where adsorption took place on the T_M_-sites, and the smallest is −199 meV on the 1H-HfSe_2_ (T_H_-site). Researchers observed that the metal sites are more energetically favorable than the T_H_-sites, and no NH_3_ adsorption occurs on the T_X_-site. In addition, researchers observed that the U-orientation is preferred to the D-orientation on the T_M_-sites. Furthermore, 1H-HfS_2_, 1H-ZrS_2_, and 1H-ZrSe_2_ are more energetically favorable for NH_3_ adsorption than 1H-HfSe_2_, 1H-HfTe_2_, and 1H-ZrTe_2_. The order of favorability is HfS_2_ > ZrSe_2_ > ZrS_2_ > HfTe_2_ (ZrTe_2_) > HfSe_2_. Conversely, NO_2_ adsorbs only on the T_X_-sites of the 1H Zr (Hf) dichalcogenides. Compared to NH_3_, NO_2_ shows a high absorption favorability, as a more negative adsorption energy is observed on all surfaces (Table 3). Instead of 1H-HfS_2_, 1H-HfTe_2_ shows high favorability to NO_2_ adsorption. Interestingly, 1H-HfTe_2_ and 1H-ZrTe_2_ shows high NO_2_ adsorption energies, in contrast to the energies of NH_3_ adsorption. The order of NO_2_ adsorption favorability is HfTe_2_ > ZrTe_2_ > ZrS_2_ > ZrSe_2_ > HfS_2_ > HfSe_2_. Although NO_2_ shows high adsorption favorability relative to NH_3_ on the 1H Zr (Hf) dichalcogenides, NH_3_ is more favorable on 1H-HfS_2_ than NO_2_, which exhibits an adsorption energy almost 200 meV larger.

Table 4 depicts the calculated E_ads_ values for NH_3_ and NO_2_ on the 1T Zr (Hf) dichalcogenides. NH_3_ shows an energetically high adsorption favorability on 1T-ZrS_2_ surfaces, with an E_ads_ value of −587 meV (T_M_-sites) and a lower value on 1T-ZrTe_2_ (−166 meV, T_H_-sites) (Figure 5). The T_M_-sites show higher NH_3_ adsorption favorability than the T_H_-sites, consistent with what was seen for NH_3_ on the 1H Zr (Hf) dichalcogenides. The preferential adsorption site of NH_3_ shifts from the T_H_-sites to the T_M_-sites for 1T-ZrS_2_ and 1T-HfSe_2_. The order of NH_3_ adsorption favorability also changes, suggesting that the surfaces of the 1H and 1T Zr (Hf) dichalcogenides behave differently. Conversely, this behavior is consistent with the 1H surfaces of 1T-HfTe_2_ and 1T-ZrTe_2_, showing high NO_2_ adsorption favorability. Also, instead of only T_X_-sites, energetically favorable NO_2_ adsorption on T_M_-sites for 1T-HfSe_2_, 1T-ZrSe_2_, and 1T-HfTe_2_ is observed. Thus, NH_3_ adsorption is more energetically favorable than NO_2_ on 1T-HfS_2_, 1T-ZrS_2_ and 1T-ZrSe_2_, while the opposite situation exists for 1T-HfSe_2_, 1T-HfTe_2_, and 1T-ZrTe_2_.

It is noteworthy that, consistently with all cases, the 1H-phase demonstrates different E_ads_ values with high adsorption favorability than the 1T-phase (Figure 5). This situation is reversed for NH_3_ on ZrS_2_, where the 1T-phase is more favorable for adsorption than the 1H-phase. Conversely, the values of E_ads_ for HfSe_2_ and HfTe_2_ are almost the same in both phases.

Next, a Bader analysis was employed to estimate the charge (ΔQ) transfer from the NH_3_ (NO_2_) to the surface of the 1T-(1H-) Zr (Hf) dichalcogenides (or vice versa), where the change is either positive or negative. The charge transfer from the molecules to the dichalcogenide’s surface is defined as positive here, whereas from the surface to the molecules is negative. Researchers noticed that, in order for a charge transfer to occur, the property of the material, the adsorption sites on the dichalcogenide’s surface, and the orientation of the gas molecules are crucial factors [54].

Researchers found that the NH_3_ that adsorbs on the 1H Zr (Hf) dichalcogenides depicts a positive charge, suggesting NH_3_ acts as a charge-donor (Table 3), which is well known experimentally. Although NH_3_ strongly adsorbs on 1H-HfS_2_ (M-sites), the maximum charge transfer (0.152 e) takes place when NH_3_ adsorbs on 1H-ZrS_2_ (H-sites). Interestingly, the adsorption distance is a maximum in this case. The fact that NH_3_ donates more charges at M-sites than at H-sites on MoS_2_ is noteworthy [35]. The charges transferred to 1H-HfSe_2_, 1H-ZrSe_2_, 1H-HfTe_2_, and 1H-ZrTe_2_ are 0.045, 0.081, 0.033, and 0.009 e, respectively. Comparatively more charge transfer occurs on the Zr-based chalcogenides compared to the Hf-based chalcogenides when adsorbed on the M-sites, while the opposite situation is seen on the H-sites. It seems to be that D-oriented NH_3_ donates more charge than the U-oriented NH_3_. Conversely, NO_2_ accepts an electron from the 1H-Zr (Hf) dichalcogenides (Table 3). This behavior is expected as NO_2_ is a well-known charge acceptor [35]. Like NH_3_, NO_2_ also depicts a high charge transfer on 1H-ZrTe_2_ (0.622 e), where even 1H-HfTe_2_ demonstrates a high NO_2_ adsorption energy. Furthermore, higher charge transfers occur on the Zr-based chalcogenides than on the Hf-based chalcogenides, when adsorption occurs on the X-sites, considering the same chalcogen. Also, the D-oriented NO_2_ accepts more charge than the U-oriented NO_2_, except for the case of 1H-ZrSe_2_.

Consistent with what was observed for the 1H surfaces, the NH_3_ adsorbed by the 1T Zr (Hf) dichalcogenides also depicts a positive charge, implying that NH_3_ is a donor (Table 4). Comparatively, charge transfer enhances for the 1H-Zr (Hf) dichalcogenides, except for 1T-ZrS_2_ and 1T-ZrSe_2_, where it decreases. Interestingly, more charge transfer occurs on the Hf-based chalcogenides compared to on the Zr-based chalcogenides, due to the NH_3_ adsorption taking place on either H-sites or M-sites, considering the same chalcogen. Also, the D-orientation shows a minimum charge transfer of 0.018 e on 1T-ZrTe_2_, and it depicts a maximum for 1H-ZrS_2_. The maximum charge transfer occurs for NH_3_ adsorption on 1T-HfS_2_, while it is a minimum on 1H-HfS_2_, suggesting NH_3_ behaves differently on 1T surfaces than 1H surfaces. Conversely, NO_2_ accepts a maximum charge on 1T-ZrTe_2_, similar to 1H-ZrTe_2_. Furthermore, charge transfer is different on the 1T and 1H surfaces. Overall, NH_3_ donates charges in the range of 0.01–0.15 e on 1H and 0.02–0.2 e on the 1T Zr(Hf) dichalcogenides. In contrast, NO_2_ accepts charge in the interval of 0.18–0.62 e on 1H and 0.05–0.62 e on the 1T Zr(Hf) dichalcogenides.

**Figure 6:**
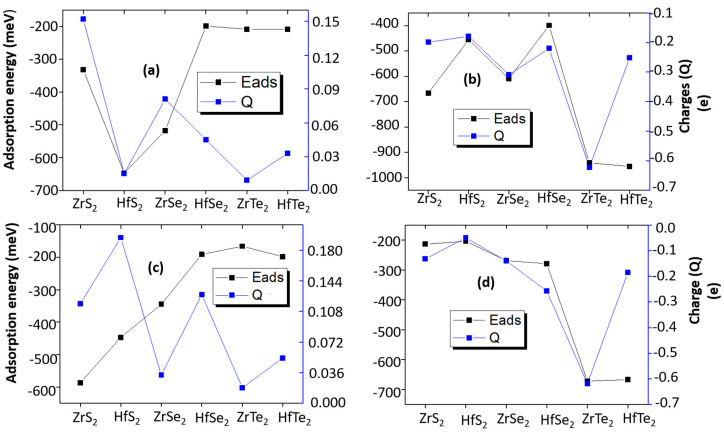
Comparisons of charge transfers with respect to adsorption energy for (**a**) NH_3__1H (**b**) NO_2__1H (**c**) NH_3__1T and (**d**) NO_2__1T.

Figure 6 depicts the correlation between E_ads_ and ΔQ for NH_3_ (NO_2_) on the Zr (Hf) dichalcogenides. The NH_3_ (NO_2_) molecules on the Zr (Hf) dichalcogenides depict a direct correlation between E_ads_ and ΔQ, where a low E_ads_ results in a low ΔQ, and vice versa. However, a different trend is seen for NH_3_ on 1H-HfS_2_ and 1H-ZrS_2_. Interestingly, the value of E_ads_ for NH_3_ on 1H-ZrS_2_ is lower than that on 1H-HfS_2_ and 1H-ZrSe_2_. However, further charge transfer occurs in the case of NH_3_ on 1H-ZrS_2_. Similarly, NH_3_ on 1T-ZrS_2_ depicts a high E_ads_, but more charge transfer occurs for NH_3_ on 1T-HfS_2_. In the case of NO_2_ adsorption, only HfTe_2_ depicts a contrary behavior. The charge transfers are quite low with respect to E_ads_, as compared with the remaining Zr (Hf) dichalcogenides.

**Figure 7:**
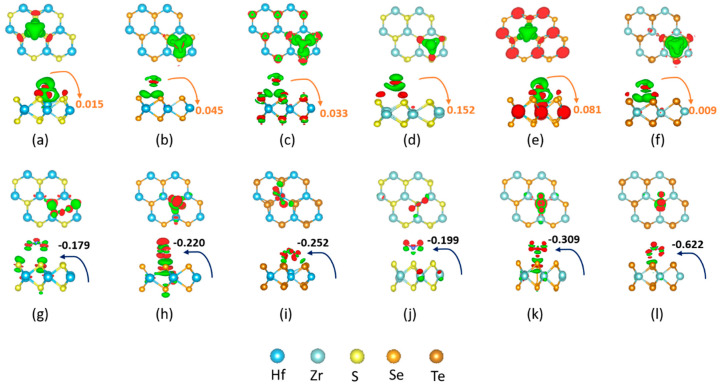
Charge density difference plots for NH_3_ (top row) and NO_2_ (bottom row) interacting with 1H: [(**a**),(**g**)] HfS_2_; [(**b**),(**h**)]HfSe_2_; [(**c**),(**i**)]HfTe_2_; [(**d**),(**j**)] ZrS_2_; [(**e**),(**k**)] ZrSe_2_; and [(**f**),(**l**)] ZrTe_2_ monolayer sheet. The isosurface is taken as 5 × 10^−3^ *e*/A3. The red or green color distribution corresponds to charge accumulation or depletion.

**Figure 8:**
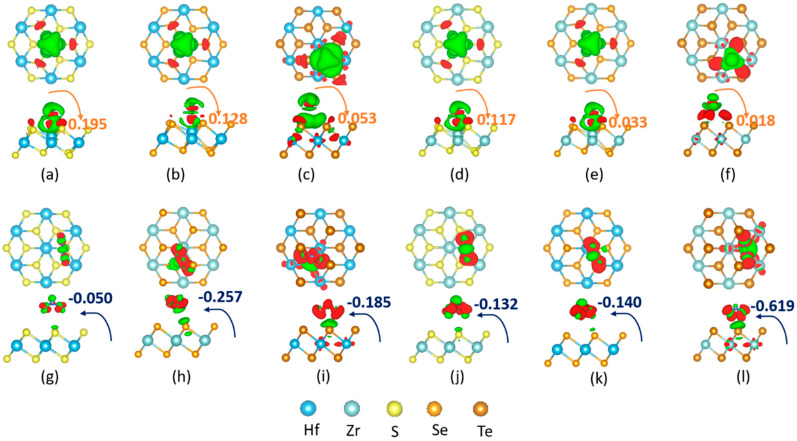
Charge density difference plots for NH_3_ (top row) and NO_2_ (bottom row) interacting with 1T: [(**a**),(**g**)] HfS_2_; [(**b**),(**h**)]HfSe_2_; [(**c**),(**i**)]HfTe_2_; [(**d**),(**j**)] ZrS_2_; [(**e**),(**k**)] ZrSe_2_; and [(**f**),(**l**)] ZrTe_2_ monolayer sheet. The isosurface is taken as 5 × 10^−3^ *e*/A3. The red or green color distribution corresponds to charge accumulation or depletion.

To gain further insight into the NH_3_(NO_2_)–TMDC interaction, we investigated the charge-transfer mechanism between them. Figure 7 and Figure 8 depict charge density difference images for NH_3_ and NO_2_ adsorbed on the 1H- (1T-) Zr (Hf) dichalcogenides. The red region shows the charge accumulation, while the green region represents the charge depletion. Different TMDCs polarize differently upon adsorption of NH_3_ (NO_2_), and the 1H surfaces show different polarizations than the 1T surfaces. Additionally, electrostatic interactions play a role in the attractive interaction. Researchers clearly observed that a charge accumulation occurs due to the adsorption of NH_3_ on either of the 1H (Figure 7) or 1T (Figure 8) surfaces, suggesting NH_3_ acts as a donor. Nevertheless, the NO_2_ adsorption shows the opposite behavior. On the 1H surfaces, NH_3_ adsorption shows a moderate charge density difference on 1H-HfS_2_ and 1H-ZrSe_2_, giving rise to a moderate interaction energy and a comparatively low charge density difference on the remaining surfaces. This explains why the former exhibits larger adsorption energies (−647 and −518 meV for NH_3_ on 1H-HfS_2_ and 1H-ZrSe_2_) than the latter (−199, −208, −332, and −208 meV for NH_3_ on 1H-HfSe_2_, 1H-HfTe_2_, 1H-ZrS_2_, and 1H-ZrTe_2_) (Table 3 and Figure 6). In contrast, the polarization increases when NO_2_, instead of NH_3_, adsorbs on the 1H surfaces. Along with moderate, a comparatively strong polarization is observed on 1H-HfTe_2_ and 1H-ZrTe_2_. Conversely, instead of 1T-ZrSe_2_, 1T-ZrS_2_ shows a moderate polarization upon adsorption of NH_3_. The remaining surfaces depict different polarizations compared to the corresponding 1H-surfaces; i.e., the NO_2_ that adsorbed on the 1T surfaces shows less polarization for all cases.

**Figure 9:**
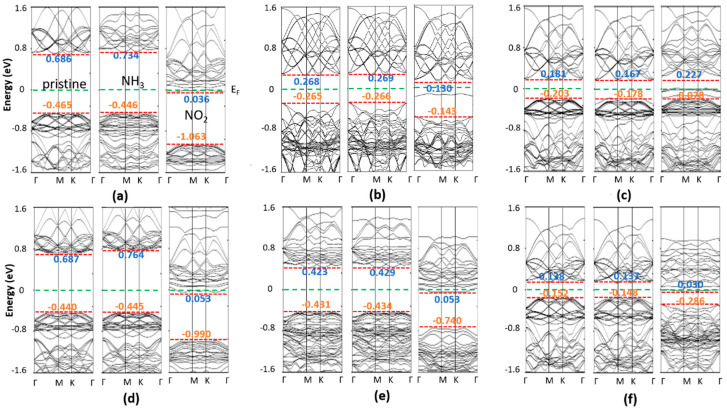
Band structures of pristine, NH_3_ and NO_2_ adsorbed monolayer 1H: (**a**) HfS_2_, (**b**) HfSe_2_, (**c**) HfTe_2_, (**d**) ZrS_2_, (**e**) ZrSe_2_, and (**f**) ZrTe_2_.

**Figure 10:**
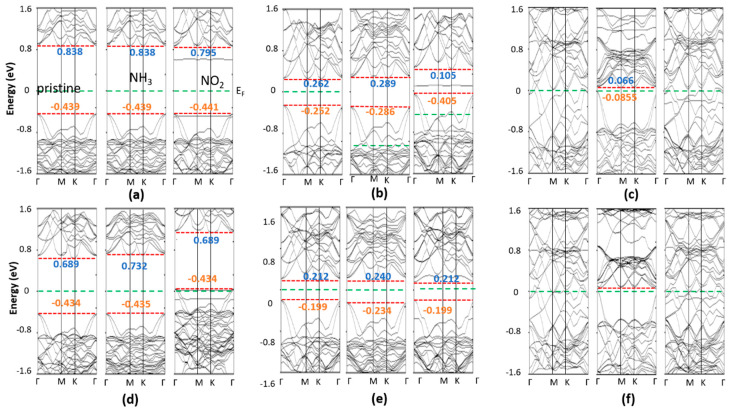
Band structures of pristine, NH_3_ and NO_2_ adsorbed monolayer 1T (**a**) HfS_2_, (**b**) HfSe_2_, (**c**) HfTe_2_, (**d**) ZrS_2_, (**e**) ZrSe_2_, and (**f**) ZrTe_2_.

The electronic properties of NH_3_ (NO_2_) adsorbed on the 2D TMDCs were examined. Here, the position of the valance and conduction bands, and the change of the Fermi level, along with the pristine 1H (1T) band positions, were considered. The calculated band structure of NH_3_ (NO_2_) adsorbed on the 1H and 1T 2D Zr (Hf) dichalcogenides are plotted in Figure 9 and Figure 10, respectively. Researchers found that the valance band maxima and conduction band minima are not at the same points for all cases, implying they are all indirect band gap materials. Researchers observed that the adsorption of NH_3_ molecules does not introduce any additional states within the band gap of the pristine 1H (1T) Zr (Hf) dichalcogenides. Furthermore, 0.001–0.04 eV changes were seen at the positions of the conduction and valance bands for most materials, which are almost insignificant and consistent with the adsorption of NH_3_ on other metal chalcogenides [35]. The conduction bands of 1H-HfS_2_, 1H-ZrS_2_, and 1T-ZrS_2_ depict an upshift of 0.048, 0.077, and 0.043 eV, respectively, from the Fermi level. All the NH_3_-adsorbed material bands features are similar to those of the pristine materials, except for 1H-HfS_2_, 1T-HTe_2_, and 1T-ZrTe_2_, and an observable conduction band degeneracy is seen for 1H-HfS_2_. Interestingly, gapless 1T-HTe_2_ and 1T-ZrTe_2_ depict band openings (~0.1 eV) with clearly separated conduction and valance bands after NH_3_ adsorption, although the band gap values are very small.

Conversely, no influence of NO_2_ adsorption on the band structures of 1T-HTe_2_ and 1T-ZrTe_2_ is seen. NO_2_ adsorption induces a state within the band gap of Zr (Hf) dichalcogenides, contrary to that happens after NH_3_ adsorption. In 1H-HfS_2_ (1H-ZrS_2_), NO_2_ induces a state that crosses the Fermi level. Other state levels appear just above E_F_. The states and conduction bands are so near (Figure 9) that the electron can easily move to-and-fro. Therefore, the estimated bandgaps of 1H-HfS_2_ and 1H-ZrS_2_ are less than those of the pristine ones. In 1H-HfSe_2_, a single state at −0.1 eV in-between the bandgap is observed with a slight downshifting of the conduction band (0.122 eV), and a comparatively large valance band shift (0.138 eV) results in bandgap enhancement. 1H-ZrSe_2_ depicts features similar to those seen for 1H-ZrS_2_; however, no conduction band expansion occurs. Two additional states cut the E_F_, where one appears below E_F_ in the case of 1H-HfTe_2_. No significant shifting in the bands occurs. Like 1H-ZrS_2_ and 1H-ZrSe_2_, the 1H-ZrTe_2_ band is just above the Fermi level, with conduction bands near to these levels, while two states cut E_F_ in 1H-HfTe_2_, and one appears just below E_F_. These states are near to the valance band. Easy to-and-fro movement of the electrons from these states to the conduction band is observed for NH_3_ adsorbed on the 1H Zr (Hf) dichalcogenides, except 1H-HfSe_2_ and 1H-HfTe_2_, where electron transfer occurs from the valance band. The NO_2_-adsorbed 1T Zr(Hf) dichalcogenides bands are identical to those of the pristine materials, but with additional band states. A single state between E_F_ and the conduction band appears in the NO_2_-adsorbed 1T-HfS_2_, while the band positions remain unchanged. Similar phenomena is observed for 1H-HfSe_2_, but with an upward shift in the band positions. Furthermore, a single state appears just below the conduction band in the NO_2_-adsorbed 1T-ZrS_2_, where a minor upward shift in the valance band results in a decrease in the band gap. The bands of 1T-ZrS_2_ shift like those of 1H-HfSe_2_, where the two states cut E_F_.

## 4. Conclusions

The molecular adsorption of NH_3_ and NO_2_ gas molecules on 1H and 1T 2D Zr (Hf) dichalcogenides (S, Se, Te) was carried out using DFT. The optimized lattice parameters of 1H were slightly smaller in all cases, compared to those of the 1T Zr (Hf) dichalcogenides. The Zr (Hf) telluride possesses semimetal features in the 1T-phase and semiconductor features in the 1H-phase. The band gaps of the Zr (Hf) dichalcogenides were found to be dependent not only of the metal species Zr and Hf but also of chalcogen, which decreases from S to Se to the Te chalcogen. Both NH_3_ and NO_2_ adsorbed exothermically on the 1H and 1T 2D Zr (Hf) dichalcogenides. Noticeably, the adsorption energy was consistently different on the two different phases of the Zr (Hf) dichalcogenides. Researchers observed that NH_3_ donates a charge to the surface, while NO_2_ accepts charge on adsorption. Moreover, a single NO_2_ molecule accepts comparatively more charge than a single NH_3_ molecule donates. Moreover, a direct correlation between the adsorption energy and charge transfer was seen, except for NH_3_ on 1H-HfS_2_ and NO_2_ on HfTe_2_. The adsorption of NO_2_ on HfS_2_, ZrS_2_, HfSe_2_, and ZrSe_2_ exhibited significant effects on the conduction and valance bands, thereby affecting the band gaps. Our results showed that the electronic properties of 2D Zr (Hf) dichalcogenides can be precisely tuned by the molecular adsorption of NH_3_ and NO_2_, which may be useful for future electronic devices.

## Figures and Tables

**Table 1:** The optimized lattice parameters (a) of Trigonal prismatic (1H) and Octahedral (1T) Zr (Hf) dichalcogenides calculated by PBE functional while keeping the distance between neighboring layers 20 Å [38,39,40,41].

Material	Trigonal Prismatic *a* (Å)	Deviation with 1T (%)	Octahedral *a* (Å)	Literature *a* (Å)		Deviation (%)	
				calc.	exp’t.	calc.	exp’t.
HfS_2_	3.51	3.04	3.62	3.66	3.63	1.09	0.27
ZrS_2_	3.54	3.01	3.65	3.69	3.66	1.08	0.27
HfSe_2_	3.64	2.41	3.73	3.78	3.75	1.06	1.61
ZrSe_2_	3.69	2.12	3.77	3.80	3.77	0.79	0
HfTe_2_	3.80	2.06	3.88	3.95		1.77	
ZrTe_2_	3.88	0.77	3.91	3.95		1.01	

***calc.:** calculated values from literature, **exp’t:** experimental values from literature* [33,34,35,36].

**Table 2:** Calculated indirect band gap values for trigonal prismatic (1H) and octahedral (1T) 2D Zr and Hf dichalcogenides (S, Se and Te).

Material	Trigonal Prismatic			Octahedral			Literature	
	Pristine	NH_3_	NO_2_	Pristine	NH_3_	NO_2_	calc.	exp’t.
HfS_2_	1.15	1.18	1.03	1.29	1.29	1.24	1.29	1.96
ZrS_2_	1.13	1.21	0.94	1.13	1.18	1.20	1.02	1.68
HfSe_2_	0.53	0.53	0.68	0.51	0.57	0.51	0.68	1.13
ZrSe_2_	0.85	0.86	0.75	0.41	0.47	0.40	0.42	1.2
HfTe_2_	0.38	0.34	0.43	0	0.10	0	0	-
ZrTe_2_	0.29	0.28	0.25	0	0.13	0	0	-

***calc.:** calculated values from literature, **exp’t:** experimental values from literature* [34,35,36,38,40].

**Table 3:** The Preferable adsorption site, orientation of gas molecule, adsorption energy (E_ads_), adsorption-distance (d) and the charge transfer (ΔQ) for NH_3_ (NO_2_) on 1H Zr (Hf) dichalcogenides.

	Gas	NH_3_					NO_2_				
Surface		Site	Orientation	E_ads_ (meV)	d(Å)	ΔQ (e)	Site	Orientation	E_ads_ (meV)	d(Å)	ΔQ (e)
HfS_2_		T_M_	U	−647	2.43	0.015	Tx	U	−456	2.02	−0.179
ZrS_2_		T_H_	D	−332	3.60	0.152	Tx	U	−666	3.24	−0.199
HfSe_2_		T_H_	U	−199	3.38	0.045	Tx	U	−399	2.20	−0.220
ZrSe_2_		T_M_	U	−518	2.49	0.081	Tx	U	−609	2.17	−0.309
HfTe_2_		T_H_	U	−208	3.56	0.033	Tx	D	−965	2.28	−0.252
ZrTe_2_		T_H_	U	−208	3.55	0.009	Tx	D	−942	2.28	−0.622

Where T_M_: metal (Hf or Zr), T_H_: Hexagon, T_X_: chalcogen (S, Se, Te), U and D refer to the orientation of NH_3_ (NO_2_).

**Table 4:** The Preferable adsorption site, orientation of gas molecule, adsorption energy (E_ads_), adsorption-distance (d) and the charge transfer (ΔQ) for NH_3_ (NO_2_) on 1T Zr (Hf) dichalcogenides.

	Gas	NH_3_					NO_2_				
Surface		Site	Orientation	E_ads_ (meV)	d(Å)	ΔQ (e)	Site	Orientation	E_ads_ (meV)	d(Å)	ΔQ (e)
HfS_2_		T_M_	U	−447	2.42	0.195	Tx	D	−204	3.30	−0.050
ZrS_2_		T_M_	U	−587	2.44	0.117	Tx	D	−214	2.66	−0.132
HfSe_2_		T_M_	U	−191	2.45	0.128	T_M_	D	−279	4.82	−0.257
ZrSe_2_		T_M_	U	−345	2.48	0.033	T_M_	U	−269	4.35	−0.140
HfTe_2_		T_H_	U	−198	3.58	0.053	T_M_	D	−667	5.12	−0.185
ZrTe_2_		T_H_	D	−166	3.87	0.018	Tx	D	−672	3.21	−0.619

Where T_M_: metal (Hf or Zr), T_H_: Hexagon, T_X_: chalcogen (S, Se, Te), U and D refer to the orientation of NH_3_ (NO_2_).
